# UK cost-effectiveness analysis of endoscopic sleeve gastroplasty versus lifestyle modification alone for adults with class II obesity

**DOI:** 10.1038/s41366-023-01374-6

**Published:** 2023-09-06

**Authors:** Jamie Kelly, Vinod Menon, Frank O’Neill, Laura Elliot, Emily Combe, Will Drinkwater, Sally Abbott, BuHussain Hayee

**Affiliations:** 1 University Hospital Southampton NHS Foundation Trust, Southampton, UK; 2 University Hospitals Coventry & Warwickshire NHS Foundation Trust, Coventry, UK; 3 University of Warwick, Coventry, UK; 4 Apollo Endosurgery UK Ltd, Knaresborough, UK; 5 FIECON Ltd, St Albans, UK; 6 Research Centre for Healthcare and Communities, Institute of Health and Wellbeing, Coventry University, Coventry, UK; 7 King’s College Hospital NHS Foundation Trust, London, UK

**Keywords:** Health policy, Obesity

## Abstract

**Background:**

Endoscopic sleeve gastroplasty (ESG) is a minimally invasive procedure that has been demonstrated in the MERIT randomised, controlled trial to result in substantial and durable additional weight loss in adults with obesity compared with lifestyle modification (LM) alone. We sought to conduct the first cost-effectiveness analysis of ESG versus LM alone in adults with class II obesity (BMI 35.0–39.9 kg/m^2^) from a national healthcare system perspective in England based on results from this study.

**Methods:**

A 6-state Markov model was developed comprising 5 BMI-based health states and an absorbing death state. Baseline characteristics, utilities, and transition probabilities were informed by patient-level data from the subset of patients with class II obesity in MERIT. Adverse events (AEs) were based on the MERIT safety population. Mortality was estimated by applying BMI-specific hazard ratios from the published literature to UK general population mortality rates. Utilities for the healthy weight and overweight health states were informed from the literature; disutility associated with increasing BMI in the class I-III obesity health states was estimated using MERIT utility data. Disutility due to AEs and the prevalence of obesity-related comorbidities were based on the literature. Costs included intervention costs, AE costs, and comorbidity costs.

**Results:**

ESG resulted in higher overall costs than LM alone but led to an increase in quality-adjusted life years (QALYs). The incremental cost-effectiveness ratio (ICER) for ESG vs LM alone was £2453/QALY gained. ESG was consistently cost effective across a wide range of sensitivity analyses, with no ICER estimate exceeding £10,000/QALY gained. In probabilistic sensitivity analysis, the mean ICER was £2502/QALY gained and ESG remained cost effective in 98.25% of iterations at a willingness-to-pay threshold of £20,000/QALY.

**Conclusion:**

Our study indicates that ESG is highly cost effective versus LM alone for the treatment of adults with class II obesity in England.

## Background

The growing obesity epidemic is a leading cause of death and disability in Europe, and the UK has one of the highest reported obesity rates in the region [1]. Data from the Health Survey for England 2021 show that one-quarter (25.9%) of adults in England are living with obesity [2]. Obesity is a major risk factor for many chronic diseases including type 2 diabetes and cardiovascular disease, and is associated with reduced quality of life and increased risk of premature death [3, 4]. Multi-component management with lifestyle modification (LM) comprising diet, physical exercise, and behavioural therapy (*±* pharmacologic treatment) is the standard first-line treatment for adults with obesity [5, 6]. However, resulting weight loss is typically modest and usually not maintained, and bariatric surgery is often needed to achieve the substantial and sustained weight reduction necessary to significantly improve general health [5, 6].

Contemporary guidelines from the American Society for Metabolic and Bariatric Surgery/International Federation for the Surgery of Obesity and Metabolic Disorders broadly recommend bariatric intervention for adults with class II obesity (body mass index [BMI] 35.0–39.9 kg/m^2^) regardless of the presence, absence, or severity of obesity-related comorbidities [5]. Notably, non-surgical treatments are described in these guidelines as being ‘ineffective’ in achieving adequate weight loss for people with class II obesity. Guidelines from the UK National Institute for Health and Care Excellence (NICE) recommend consideration of bariatric intervention alongside an intensive ‘Tier 3’ weight management programme delivered by a multidisciplinary team for adults with class II obesity [6]. To be eligible, patients with class II obesity are required to have at least one obesity-related comorbidity and non-surgical weight management measures not achieved or maintained clinically beneficial weight loss. However, the guideline recommendations on bariatric surgery were published almost a decade ago and a comprehensive review/update is underway [7]. Several bariatric procedures are available and used in UK clinical practice (including Roux-en-Y gastric bypass, laparoscopic sleeve gastrectomy, and gastric banding) [8]. However, these are invasive, associated with risk of post-operative complications, and hesitancy about undergoing surgery from the perspective of patients is common [5, 9, 10]. Further, only 20% of procedures recorded in the UK National Bariatric Surgery registry during 2013-2018 were performed as day cases, with patients typically requiring inpatient admission for 2 to 3 days [8].

Obesity disproportionately affects the most deprived communities in England [2, 8]. Desogus et al, estimated that 7.8 million people in England would have met NICE’s eligibility criteria for bariatric surgery in 2014 [11]. However, it is widely acknowledged that access to bariatric services within the NHS is heavily rationed by clinical commissioning groups in England and fewer than 5000 NHS-funded procedures were recorded in the National Bariatric Surgery Registry for 2019, suggesting that demand far outstrips capacity [8, 12, 13].

Endoscopic sleeve gastroplasty (ESG) is a minimally invasive procedure that uses full-thickness suturing to reduce gastric capacity and delay emptying [14]. Multiple meta-analyses have demonstrated that ESG is an effective and safe method of weight loss for people with obesity [15, 16]. The MERIT randomised, controlled trial (RCT) is the only RCT conducted to date that has evaluated the effectiveness and safety of ESG, which was performed in the study using the first and only approved ESG device (Overstitch^TM^*;* Apollo Endosurgery, Austin Tx, US) [14]. In a cohort of 209 adults with class I or class II obesity (BMI 30.0–39.9 kg/m^2^) who had a history of unsuccessful attempts at weight loss with conservative methods, ESG alongside LM resulted in rapid and sustained additional excess weight loss versus LM alone, as well as improvements in obesity-related comorbidities. Use of ESG for the treatment of obesity is the subject of an upcoming review by NICE via its interventional procedure appraisal programme [17]. Whilst numerous cost-effectiveness analyses of other bariatric interventions for obesity have been previously published [18, 19], none have evaluated ESG. We aimed to provide the first cost-effectiveness analysis of ESG in the UK by leveraging data from the MERIT RCT. The findings from this study are expected to be relevant for future clinical and economic decision making in the UK, and could potentially inform the ongoing NICE obesity guidelines update.

## Methods

A cost-utility analysis was conducted for a UK (England) population in line with the NICE reference case [20]. This included adopting a National Health Service (NHS) and personal social services perspective for costs, measuring health benefits with quality-adjusted life years (QALYs), using a lifetime time horizon, and applying a 3.5% annual discount rate for costs and health effects. The incremental cost-effectiveness ratio (ICER) was estimated and cost effectiveness evaluated using the lower bound of the £20,000–30,000/QALY willingness-to-pay threshold range adopted by NICE [21]. Reporting was aligned with the Consolidated Health Economic Evaluation Reporting Standards (CHEERS) checklist (Table S1) [22].

The analysis considered the cost effectiveness of ESG alongside LM versus LM alone based on the MERIT RCT with the model population limited to patients with class II obesity (i.e., who could potentially be eligible for ESG per current international and UK guidelines). In this MERIT subgroup (*n* = 115), the mean age at baseline was 42 years (range 20–64), mean BMI was 37.5 (range 34.33–39.91), and 14.0% of participants were male; a total of 58.1% had hypertension, 33.0% had type 2 diabetes, and 20.0% had sleep apnoea [23].

The comparison of ESG with LM alone in the model is relevant as MERIT is the only RCT conducted to date that has evaluated the effectiveness and safety of ESG and represents the most robust source of clinical evidence on the procedure. In the model, LM reflected Tier 3 weight management services recommended in NICE clinical guidelines [6]. This is typically administered over a 2-year duration and comprises a specialist physician, a dietician, a specialist nurse, and a clinical psychologist with access to physical therapy [24]. The ESG procedure was assumed to be performed using the same device (i.e., Overstitch™) and in the same outpatient setting as the MERIT study.

Authors JK, VM, SA and BH provided expert clinical advice to validate the model structure, inputs/assumptions, and plausibility of the results. Given the technical modelling nature of the study, patient group input was not solicited during model development, though results were presented to Obesity UK.

### Model

**Fig. 1:**
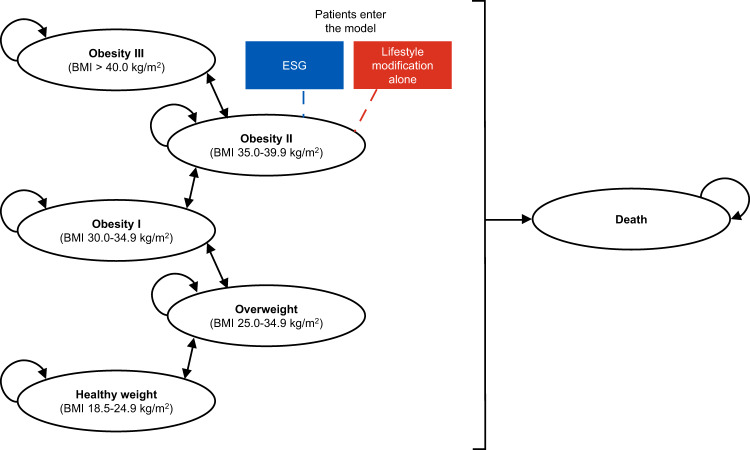
Model schematic. Patients enter the model in the obesity II health state and receive either ESG or lifestyle modification alone. Based on model transition probabilities, in each model cycle patients can either move to an adjacent obesity health state (or the death state) or remain in the same obesity health state. BMI body mass index, ESG endoscopic sleeve gastroplasty.

A de novo 6-state Markov model was developed in Microsoft® Excel which included 5 BMI-based health states and an absorbing death state (Fig. 1). A simulated cohort of 1000 patients entered the model in the class II obesity state; patients could transition to the other model states depending on changes to weight and risk of death over the model time horizon (100 years minus the mean age of model patients at baseline). A cycle length of 6 months was used for the first year in order to reflect the immediate weight loss observed with ESG and annual cycles were used thereafter. A half-cycle correction was applied to account for the fact that events and transitions could occur at any point during the cycle.

### Clinical parameters

**Fig. 2:**
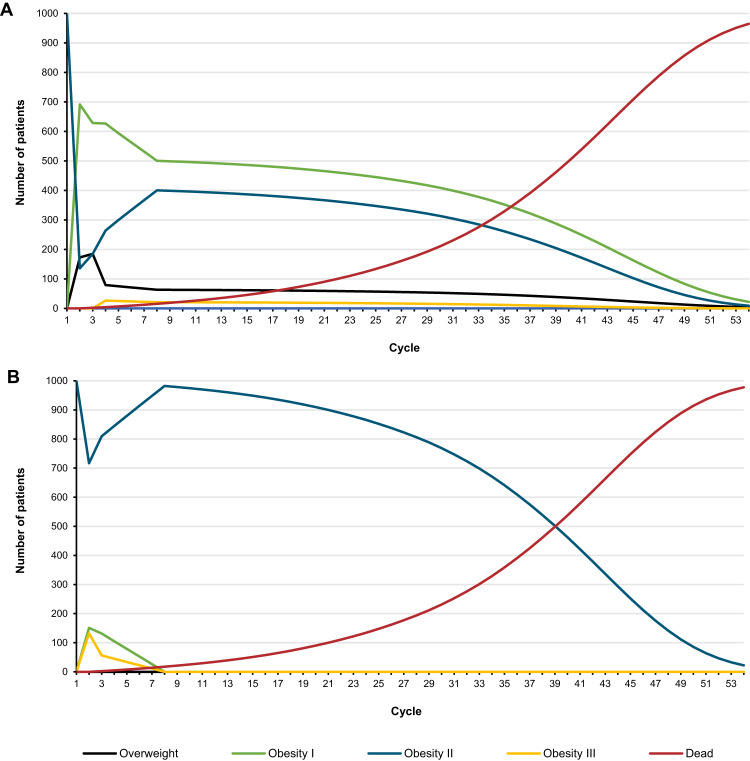
Health state transitions for ESG and lifestyle modification alone over the model time horizon. The proportion of model patients in each health state in each cycle over the model time horizon is shown for ESG (**A**) and lifestyle modification alone (**B**). The healthy weight health state is not included as no patients are projected to enter this health state at any timepoint over the model time horizon. ESG endoscopic sleeve gastroplasty, LM lifestyle modification.

Model clinical inputs and parameter values are summarised in Table 1 and Fig. 2.

**Table 1:** Summary of model clinical inputs.

Parameter		Parameter value	Source
Baseline characteristics			
Age (years), mean (SE)		46 (N/A)	[23]
Male sex, % (SE)^a^		14 (3)	
Incidence of severe adverse event^b^			[9]
Abdominal abscess, % (SE)	ESG	1.18 (N/A)	
	LM alone	0.00 (N/A)	
Upper gastrointestinal bleed, % (SE)	ESG	1.18 (N/A)	
	LM alone	0.00 (N/A)	
Malnutrition, % (SE)	ESG	1.18 (N/A)	
	LM alone	0.00 (N/A)	
BMI-specific mortality risk by health state^c^			
Healthy weight, hazard ratio (SE)		1.00 (reference)	[27]
Overweight, hazard ratio (SE)		1.00 (0.03)	
Obesity I, hazard ratio (SE)		1.12 (0.01)	
Obesity II, hazard ratio (SE)		1.36 (0.01)	
Obesity III, hazard ratio (SE)		1.88 (0.03)	
Comorbidity prevalence by health state^d^			
Type 2 diabetes, % (SE)	Healthy weight	2.30 (0.46)	[59]
	Overweight	6.20 (1.24)	
	Obesity I	13.20 (2.64)	
	Obesity II	18.60 (3.72)	
	Obesity III	25.70 (5.14)	
Hypertension, % (SE)	Healthy weight	45.00 (9.00)	[30]
	Overweight	67.00 (13.40)	
	Obesity I	79.00 (15.80)	
	Obesity II	79.00 (15.80)	
	Obesity III	87.00 (17.40)	
Sleep apnoea, % (SE)	Healthy weight	0.20 (0.04)	[31]
	Overweight	0.51 (0.10)	
	Obesity I	0.51 (0.10)	
	Obesity II	0.51 (0.10)	
	Obesity III	4.67 (0.93)	
Gastro-oesophageal reflux disease, % (SE)	Healthy weight	11.17 (2.23)	[60]
	Overweight	21.13 (4.23)	
	Obesity I	26.69 (5.34)	
	Obesity II	26.58 (5.32)	
	Obesity III	26.58 (5.32)	
Non-alcoholic fatty liver disease	Healthy weight	0.09 (0.02)	[61]
	Overweight	0.34 (0.07)	
	Obesity I	0.72 (0.14)	
	Obesity II	0.72 (0.14)	
	Obesity III	0.72 (0.14)	

*BMI* body mass index, *ESG* endoscopic sleeve gastroplasty, *LM* lifestyle modification.

^a^ Data for the subset of patients with class II obesity enroled in the MERIT study.

^b^ Data for the MERIT safety population comprising all patients who underwent ESG. Standard error is not applicable to the estimated incidence of individual adverse events, as these are multiplied separately by the cost and disutility of each adverse event to determine the total cost and total disutility of each adverse event for each treatment. These totals were then varied in one-way sensitivity analysis with an assumed standard error of ±20%.

^c^ BMI-specific all-cause mortality hazard ratio reported by Bhaskaran et al., [27] was <1.00 (0.94) for the overweight health state. This would not have been appropriate for use in the model as hazard ratios were applied to general population mortality rates and would therefore result in lower mortality for the overweight health state than for the general population. As such, the hazard ratio for the overweight health state was assumed to be 1.00 to match the healthy weight health state (the reference group).

^d^ See Tables S5, S6, and S7 for further details on the calculation of comorbidity prevalence rates for the model health states; some prevalence estimates are the same for different model health states as the source publication did not report prevalence according to the same BMI groupings used to define our model health state.

Model baseline characteristics (age and sex) and BMI group data required for the calculation of health state transition probabilities at 6 months, 1 year, and 2 years were based on patient-level data for the subgroup of patients with class II obesity from the MERIT study [23]. The last observation carried forward approach was used to impute missing BMI data at each timepoint, providing the last observation was ≤10 weeks before the timepoint (patients with last observations >10 weeks before the timepoint were excluded). In the first year where 6-month cycles were used, in the event of missing data at week 26 (6 months), values were taken in order of preference from observations at week 24, week 30, or week 16, which were all monitored visits and considered sufficient to capture weight loss associated with ESG. The MERIT study had a 2-year follow-up duration with available data for LM alone limited to the first year before patients randomised to LM were permitted to cross over and receive an ESG. Consequently, assumptions were required to extrapolate transition probabilities over the remainder of the model time horizon. Weight loss was assumed to plateau after 2 years (with BMI remaining constant thereafter) for 80% of model patients receiving ESG. To account for the potential of weight regain following ESG, the remaining 20% of patients receiving ESG were assumed to gradually return to baseline BMI by 5 years based on a recent systematic review and meta-analysis of studies assessing weight regain following bariatric surgery [25]. Weight regain was assumed to occur in all patients receiving LM after 1 year with BMI gradually returning to baseline BMI by 5 years, consistent with the approach taken in NICE’s appraisal of liraglutide for the management of obesity [26].

Adverse events (AEs) included in the model were based on the incidence of any severe AE that occurred in the MERIT safety population comprising all study participants [14]. This resulted in the inclusion of abdominal abscess, upper gastrointestinal bleed, and malnutrition AEs for ESG, and no AEs for LM.

The MERIT study included a relatively small sample size, and no deaths were observed during the 2-year follow-up period. Mortality (Fig. S1) was therefore estimated by applying BMI-specific mortality risks from a large UK population-based cohort study identified in a pragmatic literature search [27] to age/sex-matched general population mortality rates for 2021 from the Office for National Statistics [28]. The MERIT study similarly did not provide sufficient data to inform model inputs on the prevalence of obesity-related comorbidities. The prevalence of comorbidities in each health state was therefore estimated using BMI-specific rates identified through a pragmatic literature search. Comorbidities included in the model were type 2 diabetes [29], hypertension [30], sleep apnoea [31], non-alcoholic fatty liver disease [32], and gastro-oesophageal reflux disease [33]. Given the limited sample size in the MERIT study, no model subgroup analyses were conducted.

### Utilities

EQ-5D is NICE’s preferred measure of health-related quality of life for informing cost-utility analyses [20]. SF-36 data were collected in MERIT and can be directly mapped to EQ-5D using a mapping algorithm such as that from Rowen et al. [34]. We therefore conducted an analysis of patient-level SF-36 data for the class II obesity MERIT subgroup using this algorithm to inform health state utility values (Table S2). A limited number of patients transitioned into the overweight health state (*n* = 28) and no patients transitioned into the healthy weight health state during study follow-up. As such, SF-36 data from MERIT was considered inadequate for deriving utility estimates for these health states; model parameter values (Table 2) were instead informed by a large UK population-based cohort study from Stephenson et al, 2021 identified in a pragmatic literature search on the association between BMI and quality of life [35]. Further, when directly mapped from the MERIT SF-36 data, the resulting utility estimates were higher than those reported by Stephenson et al, (Table S2), likely due to the ceiling effect [36]. Therefore, in line with NICE technical guidance [37], a linear mixed-effects model was used to estimate the incremental disutility associated with increasing BMI in the obesity I-III health states (Table S3). These disutilities were applied to the overweight health state utility value taken from Stephenson et al, to derive health state utility values for the obesity I-III health states (Table 2 and Table S4).

**Table 2:** Summary of model cost and utility inputs.

	Parameter	Parameter value	Source
Cost inputs	Intervention costs		
	ESG device and procedure costs^a^	£4287.00	[62]
	Lifestyle modification costs^b,c^	£224.85	[41, 42]
	Adverse event costs (per event)^d^		
	Abdominal abscess	£821.91	[42]
	Upper gastrointestinal bleed	£1786.28	[42]
	Malnutrition	£1988.00	[42]
	Comorbidity costs (annual cost per patient)		
	Type 2 diabetes	£2129.00	[29]
	Hypertension^e^	£36.19	[43–46]
	Sleep apnoea^f,g^	£1537.42	[31]
	Gastro-oesophageal reflux disease	£389.20	[33]
	Non-alcoholic fatty liver disease^g^	£397.53	[32]
Utility inputs	Health state utility values, mean (SE)		
	Healthy weight	0.85 (0.001)	[35]
	Overweight	0.81 (0.002)	[35]
	Obesity I^h^	0.78 (0.030)	[23, 34, 35]
	Obesity II^h^	0.70 (0.030)	[23, 34, 35]
	Obesity III^h^	0.61 (0.040)	[23, 34, 35]
	Adverse event disutility values, mean (SE)^d^		
	Abdominal abscess	0.13 (N/A)	[40]
	Upper gastrointestinal bleed	0.15 (N/A)	[38]
	Malnutrition	0.08 (N/A)	[39]
	Total adverse event disutility values, mean (SE)^d^		
	ESG	0.004 (0.001)	[40]
	Lifestyle modification alone	0.000 (0.000)	[40]

All costs are 2020/21 values.

*ESG* endoscopic sleeve gastroplasty.

^a^ Including device costs, pre-operative assessment, surgeon/assistant time, anaesthetic, post-operative gastroscopy, and post-discharge medication.

^b^ See Table S8 for a breakdown of individual cost components, calculations, and sources.

^c^ Annual cost applied to both the ESG and lifestyle modification alone groups for the duration of the model horizon.

^d^ One-off ESG procedure-related adverse event costs and disutilities applied in cycle 1 only. Standard error is not applicable to individual adverse event disutilities, as these are applied in the model as a total treatment-related adverse event disutility; this total was then varied in one-way sensitivity analysis with an assumed standard error of ±20%.

^e^ See Table S9 for a breakdown of cost components, calculations, and sources.

^f^ Average (mean) of reported costs for HRG codes DZ18D, DZ18E, DZ18F and DZ18G.

^g^ Costs inflated from 2019 values (sleep apnoea) and 2016 values (non-alcoholic fatty liver disease) to 2020/21 values.

^h^ Health state utility estimate calculated by applying the disutility generated from a linear mixed-effects regression model to the overweight health state utility value (0.81) reported by Stephenson et al. [35].

Disutilities were also applied for ESG-related AEs, with values for abdominal abscess, upper gastrointestinal bleed, and malnutrition (Table 2) identified through a pragmatic literature search [38–40]. Given the one-off nature of the procedure, these disutilities were applied in cycle 1 only.

To avoid double counting, comorbidity-associated disutility was assumed to be already captured in the BMI-based health state utility values.

### Costs

Costs included in the model reflect intervention costs for both ESG and LM, costs associated with the management of AEs, and costs associated with treatment of obesity-related comorbidities (Table 2). These were based on 2020/21 unit costs where possible; older costs were inflated to 2020/21 values using the NHS Cost Inflation Index [41].

ESG costs were based on the cost of the device and hospital costs associated with delivery of the procedure. Costs for LM were applied to both treatment groups and were based on Tier 3 weight management, including healthcare professional visits, with cost categories (GP consultation, nurse consultation, dietician consultation, specialist consultation, consultation, and blood count) and frequency of visits taken from NICE’s appraisal of liraglutide [26]. Costs for clinical psychologist visits were also incorporated based on feedback from the clinical expert authors that these are routinely offered in Tier 3 weight management services. The cost of each component was sourced from Personal Social Services Research Unit 2021 unit costs and NHS England 2020/21 reference costs as applicable [41, 42].

As the model does not capture subsequent obesity treatment costs (e.g., bariatric procedures for eligible patients in whom treatment does not result in adequate or durable weight loss), LM costs were assumed to be incurred in both treatment groups for the duration of the model horizon. Although it is expected that a proportion of patients will not be compliant with LM medical advice about lifestyle and dietary changes over the duration of the intervention, a 100% compliance rate was assumed in the absence of robust data.

Costs for the management of obesity-related comorbidities were based on annual costs identified through a pragmatic literature search [29, 31–33, 43–46]. These annual costs were combined with the previously described comorbidity prevalence rates to estimate the total comorbidity cost for each treatment per health state per model cycle (Table S10). Costs for the management of severe AEs were applied as one-off costs in cycle 1 and sourced from the National Cost Collection 2020/21 [42].

### Sensitivity and scenario analyses

Deterministic one-way sensitivity analyses (OWSA) were conducted for each model parameter across ranges equal to the 95% confidence intervals. These were mostly calculated using a standard error of ±20% of the mean value for each parameter. Exceptions were health state utilities for which the values were sourced from the literature (for the healthy weight and overweight health states) and calculated (for the remaining health states), and the mortality hazard ratio for which the standard error was calculated. Results were plotted on a Tornado diagram to identify key drivers of cost effectiveness.

A probabilistic sensitivity analysis was conducted using 10.000 iterations to characterise overall uncertainty in the cost-effectiveness results, with values for each parameter simultaneously drawn from their individual uncertainty distribution. Results were plotted on an incremental cost-effectiveness plane scatter plot to visualise uncertainty and a cost-effectiveness acceptability curve was generated to show the probability of ESG being cost effective over a range of willingness-to-pay thresholds (£0–50,000/QALY). A full list of model parameters including uncertainties and distributions is provided in the supplementary materials.

Scenario analyses were conducted to explore structural uncertainty related to important model assumptions/inputs including use of alternative long-term BMI extrapolations. Additional scenario analyses included use of general population mortality rates without adjustment to account for the impact of BMI on mortality risk (all BMI-specific mortality HRs set to 1), use of an alternative mapping algorithm from Ara and Brazier, 2008 [47], and with all health state utility estimates based on values reported by Stephenson et al.

A threshold analysis was conducted to estimate how high total ESG device/procedure costs would need to be for the ICER to exceed the £20,000/QALY willingness-to-pay threshold.

## Results

In the base-case analysis, ESG was associated with £3024 higher costs than LM alone but resulted in 0.31 additional life years and 1.23 additional QALYs, leading to an ICER of £2453/QALY gained (Table 3) which falls well below a willingness-to-pay threshold of £20,000/QALY. The relatively low incremental cost with ESG is attributable to ESG intervention and AE costs being partly offset by savings in comorbidity costs.

**Table 3:** Deterministic base-case and key scenario analysis results.

	Costs (£)	Life years	QALYs	ICER (£/QALY)
Base-case results				
ESG	19,558	21.257	15.909	
LM alone	16,534	20.943	14.676	
Incremental (ESG vs LM)	3024	0.314	1.233	2453
Scenario 1: Alternative BMI extrapolation for ESG (BMI plateau following end of the trial observation period for 100% patients)				
ESG	19,321	21.324	16.140	
LM alone	16,534	20.943	14.676	
Incremental (ESG vs LM)	2787	0.381	1.464	1903
Scenario 2: Alternative BMI extrapolation for ESG (30% patients return to baseline BMI by year 5)				
ESG	19,681	21.222	15.789	
LM alone	16,534	20.943	14.676	
Incremental (ESG vs LM)	3147	0.279	1.113	2828
Scenario 3: Alternative BMI extrapolation for both ESG and LM (BMI plateau following end of the trial observation period for 100% patients)				
ESG	19,321	21.324	16.140	
LM alone	16,480	20.959	14.788	
Incremental (ESG vs LM)	2840	0.366	1.352	2101
Scenario 4: No health state BMI mortality risk adjustment applied to general population mortality estimates (all HRs set to 1)				
ESG	19,977	21.772	16.279	
LM alone	17,221	21.772	15.256	
Incremental (ESG vs LM)	2757	0.000	1.023	2696
Scenario 5: All health state utility values from Stephenson et al, 2021 [35]				
ESG	19,558	21.257	15.649	
LM alone	16,534	20.943	15.318	
Incremental (ESG vs LM)	3024	0.314	0.331	9134
Scenario 6: Use of alternative SF-36 to EQ-5D mapping algorithm from Ara and Brazier, 2008 [47]				
ESG	19,558	21.257	15.625	
LM alone	16,534	20.943	14.259	
Incremental (ESG vs LM)	3024	0.314	1.365	2215

*BMI* body mass index, *ESG* endoscopic sleeve gastroplasty, *HR* hazard ratio, *ICER* incremental cost-effectiveness ratio, *LM* lifestyle modification, *QALY* quality-adjusted life year.

In the probabilistic sensitivity analysis, incremental costs and QALYs from the 10,000 model iterations are shown on the incremental cost-effectiveness plane (Fig. S2) and resulted in a mean ICER of £2502/QALY gained, consistent with the base-case analysis. Further, ESG remained cost effective in 98.25% of iterations at a willingness-to-pay threshold of £20,000/QALY gained. The cost-effectiveness acceptability curve (Fig. S3) showed that with the base-case model inputs, ESG is likely to be cost effective at a willingness-to-pay threshold above £3000/QALY gained.

**Fig. 3:**
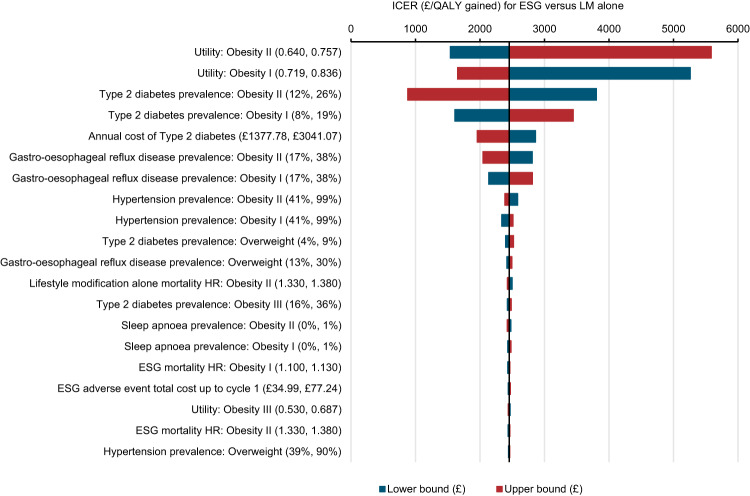
Deterministic one-way sensitivity analysis results Tornado plot. The Tornado plot generated in one-way sensitivity analysis shows the impact on the ICER of changing individual model input parameters their lower and upper bound 95% confidence intervals (shown in parentheses next to each parameter on the plot). Given the negligible impact most parameters had on the ICER the plot is limited to the 20 most impactful model parameters for brevity. ESG endoscopic sleeve gastroplasty, HR hazard ratio, QALY quality-adjusted life year.

Results from the OWSA (Fig. 3) demonstrate that the base-case analysis was broadly insensitive to changes in individual input parameters with resulting ICERs ranging from £1540 to £5585/QALY gained. The parameters that had the greatest impact on the ICER were the health state utility values and prevalence of type 2 diabetes in both the obesity I and II health states. For the remaining parameters, the OWSA showed tight intervals around the base-case estimate.

Results from scenario analyses (Table 3 and Table S14) also consistently showed ESG to be cost effective versus LM alone. Use of a more conservative long-term weight loss extrapolation for ESG with 30% patients returning to baseline BMI by 5 years resulted in a modest increase to the ICER, and even in scenarios with highly conservative weight regain assumptions for ESG (with up to 70% patients returning to baseline BMI by 5 years), the resulting ICER estimates were consistently <£6000/QALY gained. Conversely, use of more optimistic extrapolations for ESG and LM alone (with BMI plateau for 100% of patients or with weight regain occurring more gradually over 10 years for ESG) resulted in modest changes to the ICER. Use of general population mortality rates without adjustment for the potential impact of BMI on mortality (all BMI-specific mortality HRs set to 1) and use of an alternative mapping algorithm to estimate health state utility values for the obesity I-III health states similarly resulted in modest changes to the ICER. Basing all health state utility estimates on the values reported by Stephenson et al, 2021 resulted in an increase of the ICER to £9134/QALY gained which was the highest ICER estimate across all of our sensitivity and scenario analysis, though this remains well below the assumed £20,000/QALY threshold.

The threshold analysis on costs associated with the ESG device/procedure showed that these costs would need to be more than five-times higher than in our base-case analysis (>£25,926) for the ICER to exceed the £20,000/QALY willingness-to-pay threshold.

## Discussion

### Key findings

Our analysis suggests that, from the perspective of a UK healthcare payer, ESG is highly cost effective compared with LM alone for treating adults with class II obesity. The base-case ICER was £2453/QALY gained, falling well below the lower bound of NICE’s typical willingness-to-pay threshold range (£20,000/QALY). Sensitivity and scenario analyses were broadly insensitive to changes in model inputs and assumptions, consistently demonstrating the cost effectiveness of ESG versus LM alone with no ICER estimate exceeding £10,000/QALY. The use of RCT evidence to inform key model inputs represents a strength of our analysis, and the consistency of results across the wide range of sensitivity and scenario analyses we conducted suggests that our overall finding that ESG is cost effective compared with LM alone is robust.

The utility estimates for the obesity I and obesity II health states and estimated prevalence of type 2 diabetes in these health states had the largest impact on the ICER in OWSA. The finding that these model inputs had among the largest impacts on the ICER is unsurprising as patients were projected to spend most of the model time horizon in these health states (ESG, obesity I; LM, obesity II) and type 2 diabetes was associated with the highest management costs of the comorbidities included in our model. The MERIT RCT demonstrated that ESG resulted in improvement in diabetes in 93% of patients with diabetes at baseline compared with 15% of patients randomised to LM alone [14]. Long-term follow-up data from the SLEEVEPASS RCT which compared the outcomes of weight loss and remission of obesity-related comorbidities after Roux-en-Y gastric bypass versus laparoscopic sleeve gastrectomy showed that approximately one-third (26% to 33%) of patients with diabetes at baseline achieved and maintained diabetes remission for 10 years following the procedure [48]. Together, these findings suggest that the substantial additional weight loss observed with ESG is likely to result in reduced rates of diabetes and associated cost savings compared with LM alone in the longer term, consistent with our model projections.

The MERIT RCT was conducted in the US where LM was based on the Mayo Clinic HEALTH Programme including a low-calorie diet plan and physical activity counselling customised for each individual [14]. Given the modest weight loss typically observed with LM, weight loss outcomes from the MERIT LM arm were considered generalisable to the UK setting where Tier 3 weight management is used. Additionally, most patients in the MERIT class II obesity subgroup had at least one obesity-related comorbidity at baseline, consistent with NICE’s current eligibility criteria for bariatric intervention [14]. Further, the mean baseline age of 42 years (range 20–64 years) and high proportion of females in our model population (86%) is broadly consistent with UK clinical practice based on data from the UK National Bariatric Surgery Registry which show that 76% of people undergoing bariatric surgery for obesity in England during 2010–2019 were female and that the average age at the time of bariatric surgery in England is approximately 46 years (range 18–61 years) [8].

Per the NICE reference case [20], only direct costs were considered in our analysis and we have not accounted for the substantial indirect costs of obesity to patients and society, such as the widely reported wage penalty which disproportionately affects women [49].

### Consistency with other studies

To the best of our knowledge this is the first published cost-effectiveness analysis of ESG. The finding that ESG is cost effective versus LM alone is broadly consistent with published UK-based economic evaluations of other bariatric procedures for severe obesity which have been consistently shown to be cost effective versus LM with interventional costs partly offset by longer-term cost savings in the management of obesity-related comorbidities [19, 50, 51]. We have also adapted our cost-effectiveness analysis to reflect the US healthcare setting with similar results (manuscript in development) [52], suggesting that our overall findings are broadly generalisable to other developed countries. Policy makers should take into account the consistency with which studies have shown that bariatric procedures including ESG represent a cost-effective use of healthcare resources compared with LM alone when determining policy on funding and use of bariatric interventions in clinical practice.

### Limitations

As is typically the case with RCT-based cost-utility analyses, long-term health effects beyond the duration of the MERIT study were extrapolated, introducing uncertainty into our analysis. Whilst durable total body weight loss over the longer term with ESG has been shown in a previous 5-year prospective cohort study by Sharaiha et al [53], recidivism following modest initial weight loss is often observed with LM alone [53–56]. Inclusion of a weight regain assumption for 20% of patients receiving ESG in the base case represents a strength of our analysis. As highlighted by Avenell et al [18], previous economic evaluations in obesity that have assumed permanent weight loss are likely to overestimate an intervention’s cost effectiveness. Notably, in scenario analyses ESG remained cost effective even with more conservative weight regain assumptions for ESG (including highly conservative scenarios with up to 70% of patients returning to baseline BMI by 5 years) and with use of shorter model time horizons.

There were insufficient data from the MERIT study for the BMI ranges informing the healthy weight and overweight health states from which to calculate health state utility values. As such, the health state utility values for these health states were informed by values reported by Stephenson et al [35]. For the remaining health states (obesity I-III), utility was estimated by applying disutilities to the overweight health state generated from a mixed-effects model. The limited number of MERIT observations from which these remaining health state utility values were calculated represents another source of uncertainty in our analysis.

There were also insufficient data from the MERIT study from which to directly estimate mortality and rates of obesity-related comorbidities. As shown by the one-way sensitivity analysis and our scenario analysis using unadjusted general population mortality rates, the impact of BMI on mortality risk was not a key driver of cost effectiveness in our model. Comorbidity rates sourced from the literature were limited in that for some of the comorbidities included in the model, prevalence rates were reported for BMI ranges spanning multiple model health states. This approach also did not allow us to explicitly model the more complex and multifactorial relationship between bariatric procedure induced weight loss and disease remission among people with pre-existing comorbidities at baseline. Additionally, cardiovascular disease is an important obesity-related comorbidity, but we did not identify any suitable data from the literature that were both compatible with our model structure and generalisable to the UK setting. Many other conditions have also been shown to be independently associated with increases in BMI [57, 58]. Inclusion of a limited number of obesity-related comorbidities in the model could have potentially biased the analysis against ESG owing to the failure to account for additional potential comorbidity cost savings.

Lastly, given our model population was already restricted to the MERIT class II obesity subgroup (*n* = 115), including only 14% male participants, we did not consider it methodologically appropriate to conduct model subgroup analyses according to additional characteristics such as sex and age which could potentially impact the cost effectiveness of ESG.

### Future research

Future research that could validate our findings and reduce uncertainty in our estimates include longer-term randomised studies of ESG from which BMI-based health state transition probabilities, comorbidity rates, and potentially even mortality could be estimated to use directly in the model or to validate values derived from the literature. Randomised studies evaluating the impact of ESG-induced weight loss on health-related quality of life with a larger set of observations than were captured in MERIT and a cost/resource use study that could provide a more precise estimate of long-term costs associated with ESG and LM would also bring additional certainty to our findings.

Distributional effects were not formally evaluated in our model, though availability of ESG which can routinely be performed as a day case procedure could potentially help to improve efficiency in NHS service delivery and reduce health inequalities. Well conducted studies providing evidence on comparative effectiveness/safety (including risk of post-procedural complications), impact on health-related quality of life, and costs/resource use (e.g., length of inpatient stay) are needed to inform robust economic evaluations of ESG versus other bariatric procedures. Given common patient concerns about risks/complications associated with surgery, incorporation of patient preferences into any such economic evaluation will also be important.

## Conclusion

Our study indicates that ESG is highly cost effective versus LM alone for the treatment of adults with class II obesity in England. In the context of rationed access to NHS-funded bariatric services for obesity, availability of an effective and safe intervention that can be routinely delivered as a day case procedure would represent a valuable addition to complement current routine practice.

### Supplementary information

Supplementary Appendix

## Data Availability

With the exception of detailed cost data for ESG which are commercially sensitive and have not been included, all relevant data generated during this study are included in this published article and its supplementary information files. The model file is not publicly available as it contains commercially sensitive data, but a copy of the model with commercially sensitive data removed is available from the corresponding author on reasonable request. A formal health economic analysis plan is not available as none was developed in advance of the study.
