# Supplementary material for: UK cost-effectiveness analysis of endoscopic sleeve gastroplasty versus lifestyle modification alone for adults with class II obesity
Source: Int J Obes (Lond). 2023 Sep 6;47(11):1161–70. doi: 10.1038/s41366-023-01374-6 (PMC10599990; doi:10.1038/s41366-023-01374-6)
Supplement: Supplementary file 1 — Supplementary Appendix [file 41366_2023_1374_MOESM1_ESM.pdf]

Supplementary Appendix

Table S1: Consolidated Health Economic Evaluation Reporting Standards (CHEERS) checklist 2
Table S2: Health state utility estimates based on MERIT study data and published literature 4
Table S3: Health state utility linear mixed-effects model results 5
Table S4: Health state utility estimates for the obesity I, II, and III model health states based on results from the linear mixed-effects model 6
Table S5: Estimated prevalence of sleep apnoea by model health state based on study by Wall et al, 2012 7
Table S6: Estimated prevalence of non-alcoholic fatty liver disease by model health state based on study by Vusirikala et al, 2020 8
Table S7: Estimated prevalence of gastro-oesophageal reflux disease by model health state based on study by Jacobson et al, 2012 9
Table S8: Estimated costs of lifestyle management 10
Table S9: Estimated costs of hypertension 11
Table S10: Estimated total comorbidity treatment cost by model health state 12
Table S11: Disaggregated base-case results: life years 13
Table S12: Disaggregated base-case results: QALYs 14
Table S13: Disaggregated base-case results: costs 15
Table S14: Full scenario analysis results 16

Figure S1: Projected mortality for patients receiving ESG or lifestyle modification alone over the model time horizon 19
Figure S2: Probabilistic sensitivity analysis results: incremental cost-effectiveness plane 20
Figure S3: Probabilistic sensitivity analysis results: cost-effectiveness acceptability curve 21

Table S1: Consolidated Health Economic Evaluation Reporting Standards (CHEERS) checklist

	CHEERS domain	Description	Location in article
1	Title	Identify the study as an economic evaluation and specify the interventions being compared.	Title
2	Abstract	Provide a structured summary that highlights context, key methods, results, and alternative analyses.	Abstract
3	Introduction: Background and Objectives	Give the context for the study, the study question, and its practical relevance for decision making in policy or practice.	Introduction
4	Health economic analysis plan	Indicate whether a health economic analysis plan was developed and where available.	Methods
5	Study population	Describe characteristics of the study population (such as age range, demographics, socioeconomic, or clinical characteristics).	Methods
6	Setting and location	Provide relevant contextual information that may influence findings.	Methods
7	Comparators	Describe the interventions or strategies being compared and why chosen.	Methods
8	Perspective	State the perspective(s) adopted by the study and why chosen.	Methods
9	Time horizon	State the time horizon for the study and why appropriate.	Methods
10	Discount rate	Report the discount rate(s) and reason chosen.	Methods
11	Selection of outcomes	Describe what outcomes were used as the measure(s) of benefit(s) and harm(s).	Methods
12	Measurement of outcomes	Describe how outcomes used to capture benefit(s) and harm(s) were measured.	Methods
13	Valuation of outcomes	Describe the population and methods used to measure and value outcomes.	Methods
14	Measurement and valuation of resources and costs	Describe how costs were valued.	Methods
15	Currency, price date, and conversion	Report the dates of the estimated resource quantities and unit costs, plus the currency and year of conversion.	Methods
16	Rationale and description of model	If modeling is used, describe in detail and why used. Report if the model is publicly available and where it can be accessed.	Methods
17	Analytics and assumptions	Describe any methods for analyzing or statistically transforming data, any extrapolation methods, and approaches for validating any model used.	Methods
18	Characterizing	Describe any methods used for estimating how the	Methods

	heterogeneity	results of the study vary for subgroups.	
19	Characterizing distributional effects	Describe how impacts are distributed across different individuals or adjustments made to reflect priority populations.	Discussion
20	Characterizing uncertainty	Describe methods to characterize any sources of uncertainty in the analysis.	Methods
21	Approach to engagement with patients and others affected by the study	Describe any approaches to engage patients or service recipients, the general public, communities, or stakeholders (eg, clinicians or payers) in the design of the study.	Methods
22	Study parameters	Report all analytic inputs (eg, values, ranges, references) including uncertainty or distributional assumptions.	Methods; Tables (1, 2, S2-10); Figures (2, S1); Additional supplementary materials[a]
23	Summary of main results	Report the mean values for the main categories of costs and outcomes of interest and summarize them in the most appropriate overall measure.	Methods; Tables (1-3, S2, S14); Figure 2
24	Effect of uncertainty	Describe how uncertainty about analytic judgments, inputs, or projections affects findings. Report the effect of choice of discount rate and time horizon, if applicable.	Methods; Discussion; Tables (3, S14); Figure 3
25	Effect of engagement with patients and others affected by the study	Report on any difference patient/service recipient, general public, community, or stakeholder involvement made to the approach or findings of the study.	Methods
26	Study findings, limitations, generalizability, and current knowledge	Report key findings, limitations, ethical, or equity considerations not captured and how these could impact patients, policy, or practice.	Results; Discussion
27	Study funding	Describe how the study was funded and any role of the funder in the identification, design, conduct, and reporting of the analysis.	Sources of funding
28	Conflicts of interest	Report authors' conflicts of interest according to journal or International Committee of Medical Journal Editors requirements.	Financial declarations

Note: Checklist published by Husereau et al, 2022 [1].

[a] Model parameter sheet provided as a standalone Microsoft® Excel file.

Table S2: Health state utility estimates based on MERIT study data and published literature

	Based on MERIT study data[a]			
**Model health state**	**Number of SF-36 observations**	**EQ-5D value (Rowen et al.)[b]**	**EQ-5D value (Ara et al.)[c]**	**Health state utility value reported by Stephenson et al.**
Healthy weight	0	N/A	N/A	0.85
Overweight	28	0.95	0.96	0.81
Obesity I	179	0.90	0.91	0.73
Obesity II	155	0.81	0.81	0.73
Obesity III	14	0.71	0.70	0.62

[a] SF-36 data from the MERIT study (class II obesity subpopulation) [2], mapped to EQ-5D using algorithms published by Rowen at al, 2009 [3] and Ara and Brazier, 2008 [4].
[b] Mapping algorithm used in the base-case analysis.
[c] Mapping algorithm explored in a model scenario analysis.

EQ-5D EuroQol five dimensions health survey, *SF-36* 36-item short form health survey.

Table S3: Health state utility linear mixed-effects model results

	Coefficient	Standard Error	P-value
Rowen et al, 2009[a]			
Intercept (Overweight)	0.92	0.03	0.00
Obesity I	-0.03	0.03	0.96
Obesity II	-0.11	0.03	0.91
Obesity III	-0.20	0.04	0.70
Ara and Brazier, 2008[b]			
Intercept (Overweight)	0.94	0.03	0.00
Obesity I	-0.04	0.03	0.07
Obesity II	-0.13	0.03	0.00
Obesity III	-0.23	0.04	0.00

Note: Results from a linear mixed-effects regression model used to assess the incremental difference between the overweight health state utility value (0.81) reported by Stephenson et al, 2021 [5] and the EQ-5D values for the obesity I, obesity II, and obesity III model health states mapped from MERIT SF-36 data using algorithms published by Rowen et al, 2009 [3] and Ara and Brazier, 2008 [4].

[a] Mapping algorithm used in the base-case analysis.
[b] Mapping algorithm explored in a model scenario analysis.

EQ-5D EuroQol five dimensions health survey, *SF-36* 36-item short form health survey.

Table S4: Health state utility estimates for the obesity I, II, and III model health states based on results from the linear mixed-effects model

Model health state	Utility estimates based on MERIT SF-36 data mapped to EQ-5D: Algorithm from Rowen et al, 2009[b]	Utility estimates based on MERIT SF-36 data mapped to EQ-5D: Algorithm from Ara and Brazier, 2008[c]	Utility reported by Stephenson et al, 2021
Obesity I	0.78	0.77	0.73
Obesity II	0.70	0.68	0.73
Obesity III	0.61	0.58	0.62

[a] SF-36 data from MERIT (class II obesity subpopulation) [2], mapped to EQ-5D using algorithms published by Rowen et al, 2009 [3] and Ara and Brazier, 2008 [4]. Health state utility estimate calculated by applying the disutility generated from the linear mixed-effects model to the overweight health state utility value (0.81) reported by Stephenson et al, 2021 [5].
[b] Mapping algorithm used in the base-case analysis.
[c] Mapping algorithm explored in a model scenario analysis.

EQ-5D EuroQol five dimensions health survey, *SF-36* 36-item short form health survey.

Table S5: Estimated prevalence of sleep apnoea by model health state based on study by Wall et al, 2012

Model health state	Total sample size	Number of cases	Estimated prevalence[b]
Healthy weight	343 543	699	0.20%
Overweight	382 472	1943	0.51%
Obesity I and Obesity II[a]	191 306	2853	0.51%
Obesity III	16 323	762	4.67%

[a] Publication by Wall et al, 2012 [6] reports data for a group defined by a BMI range covering both the obesity I and obesity II model health states.
[b] Calculated as proportion of cases among the total sample.

Table S6: Estimated prevalence of non-alcoholic fatty liver disease by model health state based on study by Vusirikala et al, 2020

Model health state	Body size phenotype category	Total sample size	Number of cases	Estimated prevalence[b]
Healthy weight	Normal weight, 0 metabolic abnormalities	1 367 321	882	0.09%
	Normal weight, 1 metabolic abnormality	223 270	377	
	Normal weight, ≥ 2 metabolic abnormalities	116 341	219	
Overweight	Overweight, 0 metabolic abnormalities	852 223	2112	0.34%
	Overweight, 1 metabolic abnormality	307 553	1437	
	Overweight, ≥ 2 metabolic abnormalities	218 120	1158	
Obesity I, Obesity II, and Obesity III[a]	Obese, 0 metabolic abnormalities	466 571	2506	0.72%
	Obese, 1 metabolic abnormality	232 795	1909	
	Obese, ≥ 2 metabolic abnormalities	226 771	2237	

[a] Publication by Vusirikala et al, 2020 [7] reports data for an overall obese group defined by a BMI range covering the obesity I, obesity II, and obesity III model health states.
[b] Calculated as proportion of total number of incident cases across reported phenotype categories among the total sample size across reported phenotype categories for each model health state.

Table S7: Estimated prevalence of gastro-oesophageal reflux disease by model health state based on study by Jacobson et al, 2012

	Number of cases	Number of controls	Total sample size	Estimated prevalence[d]
Healthy weight[a]	776	6173	6949	11.17%
Overweight[b]	959	3580	4539	21.13%
Obesity I	458	1258	1716	26.69%
Obesity II and Obesity III[c]	214	591	805	26.58%

[a] Total mild/moderate/severe cases and controls reported across BMI groups < 22.00 kg/m^2, 20.00-22.49 kg/m^2, and 22.50-24.99 kg/m^2.
[b] Total mild/moderate/severe cases and controls reported across BMI groups < 25.00-27.49 kg/m^2 and 27.50-29.99 kg/m^2.
[c] Publication by Jacobson et al, 2012 [8] reports data for a group defined by a BMI range covering both the obesity II and obesity III model health states.
[d] Calculated as the proportion of cases among total observations.

Table S8: Estimated costs of lifestyle management

	Annual frequency per patient	Unit cost (£)	Annual cost per patient (£)	Cost source
GP consultation[a]	2	39.23	78.46	[9]
Nurse consultation[b]	4	13.75	55.00	[9]
Dietician consultation[c]	3	10.57	31.70	[9]
Specialist consultation[d]	2	27.32	54.63	[9]
Blood count	2	2.53	5.06	[10]
Clinical psychologist consultation[e]	2	32.50	65.00	[10]
Total			289.85	

Note: All costs are 2020/21 values. With the exception of clinical psychologist consultations which were incorporated based on feedback from the clinical expert authors, the cost components and their annual frequencies were based on the approach taken in NICE's appraisal of liraglutide for the treatment of obesity [11].

[a] Per surgery consultation lasting 9.22 min, with qualification costs, including direct care staff costs.
[b] Band 6 nurse: £55/h, 15 min duration assumed.
[c] Band 5 dietitian: £41/h, 15 min duration assumed.
[d] Band 6 dietician: £53/h, 15 min duration assumed.
[e] Band 7 clinical psychologist: £65/h, 30 min duration assumed.

GP general practitioner.

Table S9: Estimated costs of hypertension

	Enalapril	Lisinopril	Perindopril	Ramipril
Cost source	[12]	[13]	[14]	[15]
Daily dose, mg	10.0	20.0	4.0	2.5
Drug tariff price, £	3.35	1.00	1.41	1.09
Tablet size associated with NHS price	5	10	4	2.5
Number of tablets in a pack	28	28	30	28
Total costs per day, £	0.24	0.07	0.05	0.04
Total costs per year, £	87.34	26.07	17.16	14.21
Mean annual cost (all medications), £[a]		36.19		

Note: All costs are 2020/21 values.
[a] Based on the approach taken in NICE's appraisal of liraglutide for the treatment of obesity [11].
NHS National Health Service.

Table S10: Estimated total comorbidity treatment cost by model health state

Model health state	Annual cost (£)[a]
Healthy weight	112.26
Overweight	247.78
Obesity I	424.33
Obesity II	538.88
Obesity III	756.90

[a] Calculated for each health state by multiplying the annual cost per patient of treating each comorbidity by the estimated prevalence of each comorbidity within each health state.

Table S11: Disaggregated base-case results: life years

Model health state	LYs ESG	LYs LM alone	Increment (ESG vs LM)	Absolute increment (ESG vs LM)	Percent absolute increment (ESG vs LM)
Healthy weight	0.000	0.000	0.000	0.000	0%
Overweight	1.689	0.000	1.689	1.689	131%
Obesity I	11.756	0.520	11.237	11.237	869%
Obesity II	7.420	20.134	-12.714	12.714	983%
Obesity III	0.408	0.289	0.119	0.119	9%
Total	21.274	20.943	0.331	0.331	26%

Note: 3.5% annual discount rate applied to costs and health effects.

ESG endoscopic sleeve gastroplasty, *LM* lifestyle modification, *QALY* quality-adjusted life year.

Table S12: Disaggregated base-case results: QALYs

	QALYs ESG	QALYs LM alone	Increment (ESG vs LM)	Absolute increment (ESG vs LM)	Percent absolute increment (ESG vs LM)
Healthy weight	0.000	0.000	0.000	0.000	0%
Overweight	1.305	0.000	1.305	1.305	39%
Obesity I	8.755	0.405	8.349	8.349	293%
Obesity II	5.613	14.094	-8.481	8.481	238%
Obesity III	0.237	0.176	0.060	0.060	6%
Total	15.909	14.676	1.233	1.233	100%

Note: 3.5% annual discount rate applied to costs and health effects.

ESG endoscopic sleeve gastroplasty, *LM* lifestyle modification, *QALY* quality-adjusted life year.

Table S13: Disaggregated base-case results: costs

	Costs ESG	Costs LM alone	Increment (ESG vs LM alone)	Absolute increment (ESG vs LM alone)	Percent absolute increment (ESG vs LM alone)
Costs by health state, £					
Healthy weight	0	0	0	0	0%
Overweight	1176	0	1176	1176	39%
Obesity I	9163	295	8868	8868	293%
Obesity II	8813	16 019	-7207	7207	238%
Obesity III	406	220	186	186	6%
Total	19 558	16 534	3024	3024	100%
Costs by cost category					
Treatment costs	10 161	5783	4378	4378	145%
Comorbidity costs	9343	10 750	-1408	1408	47%
Adverse event costs	54	0	54	54	2%
Total	19 558	16 534	3024	3024	100%

Note: All costs are 2020/21 values; 3.5% annual discount rate applied to costs and health effects.
ESG endoscopic sleeve gastroplasty, *LM* lifestyle modification.

Table S14: Full scenario analysis results

	Costs (£)	Life years	QALYs	ICER (£/QALY)
Base-case results				
ESG	19 558	21.257	15.909	
LM alone	16 534	20.943	14.676	
Incremental (ESG vs LM)	3024	1.233	2.453	2453
Scenario 1: Alternative extrapolation for ESG (BMI plateau following end of trial observation period)				
ESG	19 321	21.324	16.140	
LM alone	16 534	20.943	14.676	
Incremental (ESG vs LM)	2787	0.381	1.464	1903
Scenario 2: Alternative extrapolation for ESG (following end of the trial observation period, 30% return to baseline BMI group by year 5)				
ESG	19 681	21.222	15.789	
LM alone	16 534	20.943	14.676	
Incremental (ESG vs LM)	3147	0.279	1.113	2828
Scenario 3: Alternative extrapolation for ESG (following end of the trial observation period, 40% return to baseline BMI group by year 5)				
ESG	19 804	21.187	15.669	
LM alone	16 534	20.943	14.676	
Incremental (ESG vs LM)	3271	0.243	0.993	3294
Scenario 4: Alternative extrapolation for ESG (following end of the trial observation period, 50% return to baseline BMI group by year 5)				
ESG	19 927	21.151	15.549	
LM alone	16 534	20.943	14.676	
Incremental (ESG vs LM)	3394	0.208	0.873	3888
Scenario 5: Alternative extrapolation for ESG (following end of the trial observation period, 60% return to baseline BMI group by year 5)				
ESG	20 051	21.116	15.428	
LM alone	16 534	20.943	14.676	
Incremental (ESG vs LM)	3517	0.173	0.753	4672
Scenario 6: Alternative extrapolation for ESG (following end of the trial observation period, 70% return to baseline BMI group by year 5)				
ESG	16 534	20.943	14.676	
LM alone	20 174	21.081	15.308	
Incremental (ESG vs LM)	3640	0.138	0.633	5754
Scenario 7: Alternative extrapolation for ESG (following end of the trial observation period, 20% return to baseline BMI group by year 10)				
ESG	19 512	21.265	15.948	
LM alone	16 534	20.943	14.676	
Incremental (ESG vs LM)	2978	0.321	1.272	2341

	Costs (£)	Life years	QALYs	ICER (£/QALY)
Scenario 8: Alternative extrapolation for ESG (following end of the trial observation period, 30% return to baseline BMI group by year 10)				
ESG	19 619	21.231	15.839	
LM alone	16 534	20.943	14.676	
Incremental (ESG vs LM)	3086	0.287	1.163	2654
Scenario 9: Alternative extrapolation for ESG (following end of the trial observation period, 40% return to baseline BMI group by year 10)				
ESG	19 727	21.197	15.729	
LM alone	16 534	20.943	14.676	
Incremental (ESG vs LM)	3194	0.253	1.054	3031
Scenario 10: Alternative extrapolation for ESG (following end of the trial observation period, 50% return to baseline BMI group by year 10)				
ESG	19 835	21.163	15.620	
LM alone	16 534	20.943	14.676	
Incremental (ESG vs LM)	3302	0.219	0.945	3495
Scenario 11: Alternative extrapolation for ESG (following end of the trial observation period, 60% return to baseline BMI group by year 10)				
ESG	19 943	21.129	15.511	
LM alone	16 534	20.943	14.676	
Incremental (ESG vs LM)	3410	0.185	0.835	4081
Scenario 12: Alternative extrapolation for ESG (following end of the trial observation period, 60% return to baseline BMI group by year 10)				
ESG	20 051	21.094	15.402	
LM alone	16 534	20.943	14.676	
Incremental (ESG vs LM)	3517	0.151	0.726	4844
Scenario 13: Alternative BMI extrapolation for ESG and LM (BMI plateau following end of trial observation period)				
ESG	19 321	21.324	16.140	
LM alone	16 480	20.959	14.788	
Incremental (ESG vs LM)	2840	0.366	1.352	2101
Scenario 14: All health state utility values from Stephenson et al, 2021 [5]				
ESG	19 558	21.257	15.649	
LM alone	16 534	20.943	15.318	
Incremental (ESG vs LM)	3024	0.314	0.331	9134
Scenario 15: Use of an alternative SF-36 to EQ-5D mapping algorithm from Ara and Brazier, 2008 [4]				
ESG	19 558	21.257	15.625	
LM alone	16 534	20.943	14.259	
Incremental (ESG vs LM)	3024	0.314	1.365	2215

	Costs (£)	Life years	QALYs	ICER (£/QALY)
Scenario 16: Use of Xu et al, 2018 [16] as an alternative source for BMI-based mortality hazard ratios				
ESG	19 083	20.657	15.475	
LM alone	15 680	19.913	13.955	
Incremental (ESG vs LM)	3403	0.744	1.520	2239
Scenario 17: No health state mortality risk adjustment applied to general population mortality estimates (all HRs set to 1)				
ESG	19 977	21.772	16.279	
LM alone	17 221	21.772	15.256	
Incremental (ESG vs LM)	2757	0.000	1.023	2696
Scenario 18: Use of 0% discount rate for costs and outcomes				
ESG	31 376	36.972	27.658	
LM alone	28 994	35.988	25.208	
Incremental (ESG vs LM)	2382	0.984	2.450	972
Scenario 19: Use of 6% discount rate for costs and outcomes				
ESG	15 497	15.861	11.877	
LM alone	12 198	15.706	11.009	
Incremental (ESG vs LM)	3299	0.156	0.869	3798
Scenario 20: Use of 20-year time horizon				
ESG	14 665	14.758	11.052	
LM alone	11 366	14.707	10.310	
Incremental (ESG vs LM)	3300	0.051	0.742	4449
Scenario 21: Use of 10-year time horizon				
ESG	10 138	8.771	6.588	
LM alone	6436	8.758	6.146	
Incremental (ESG vs LM)	3703	0.013	0.441	8390

BMI body mass index, *ESG* endoscopic sleeve gastroplasty, *ICER* incremental cost-effectiveness ratio, *LM* lifestyle management, *LY* life year, *QALY* quality-adjusted life year.

Figure S1: Projected mortality for patients receiving ESG or lifestyle modification alone over the model time horizon

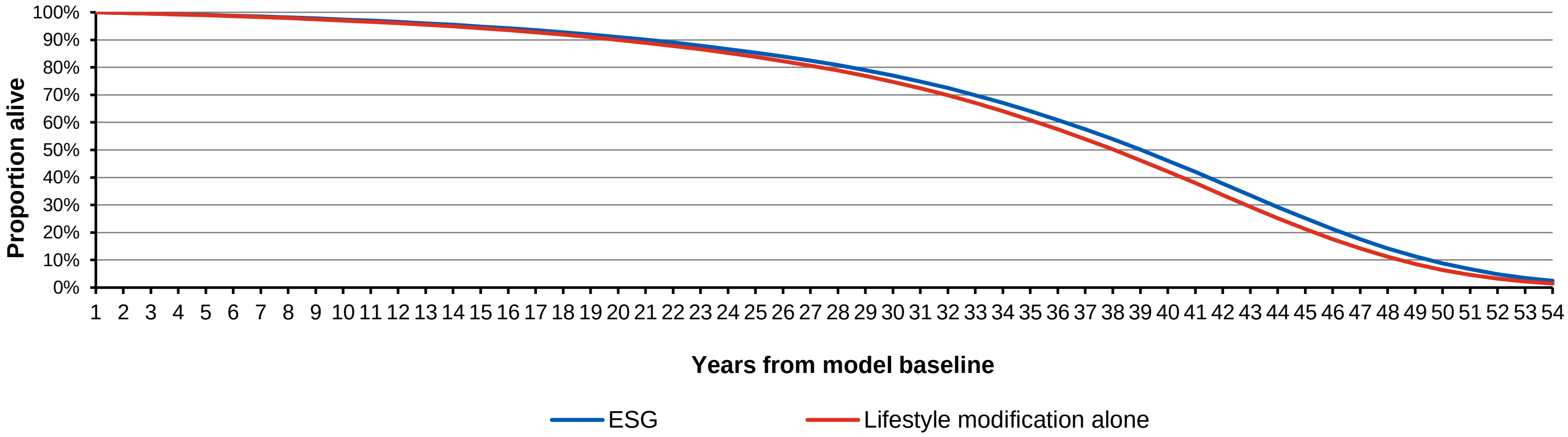

ESG endoscopic sleeve gastroplasty.

Figure S2: Probabilistic sensitivity analysis results: incremental cost-effectiveness plane

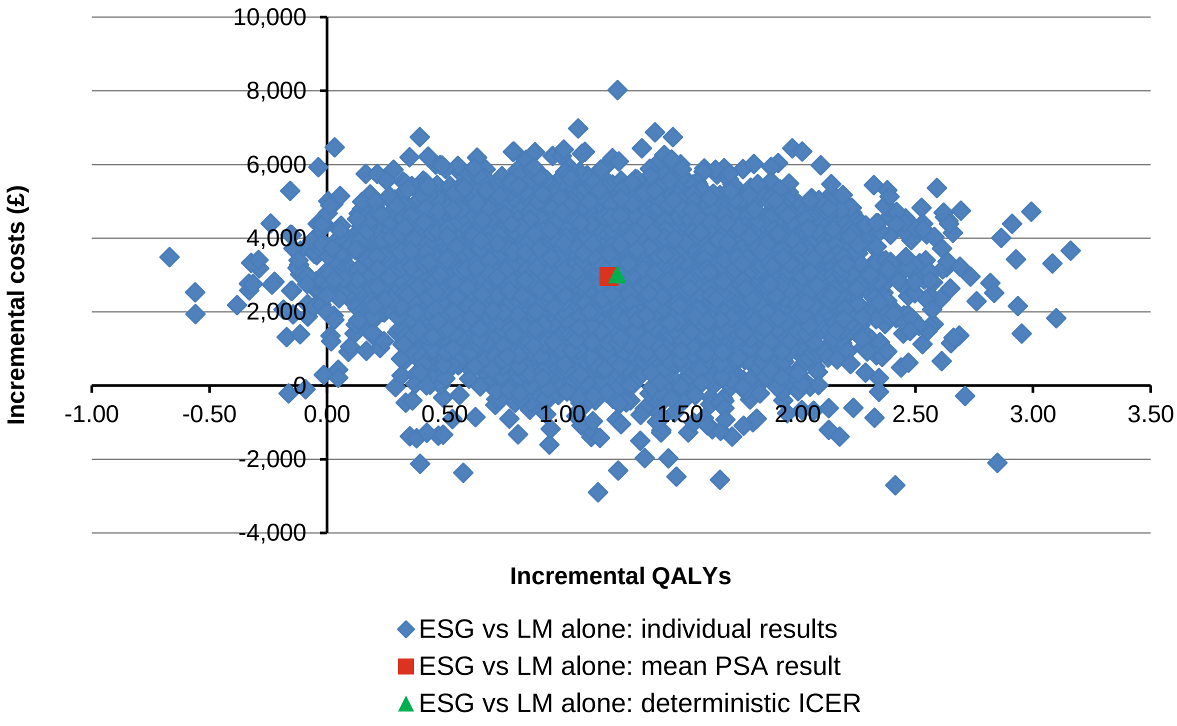

ESG endoscopic sleeve gastroplasty, *ICER* incremental cost-effectiveness ratio, *LM* lifestyle modification, *PSA* probabilistic sensitivity analysis, *QALY* quality-adjusted life year.

Figure S3: Probabilistic sensitivity analysis results: cost-effectiveness acceptability curve

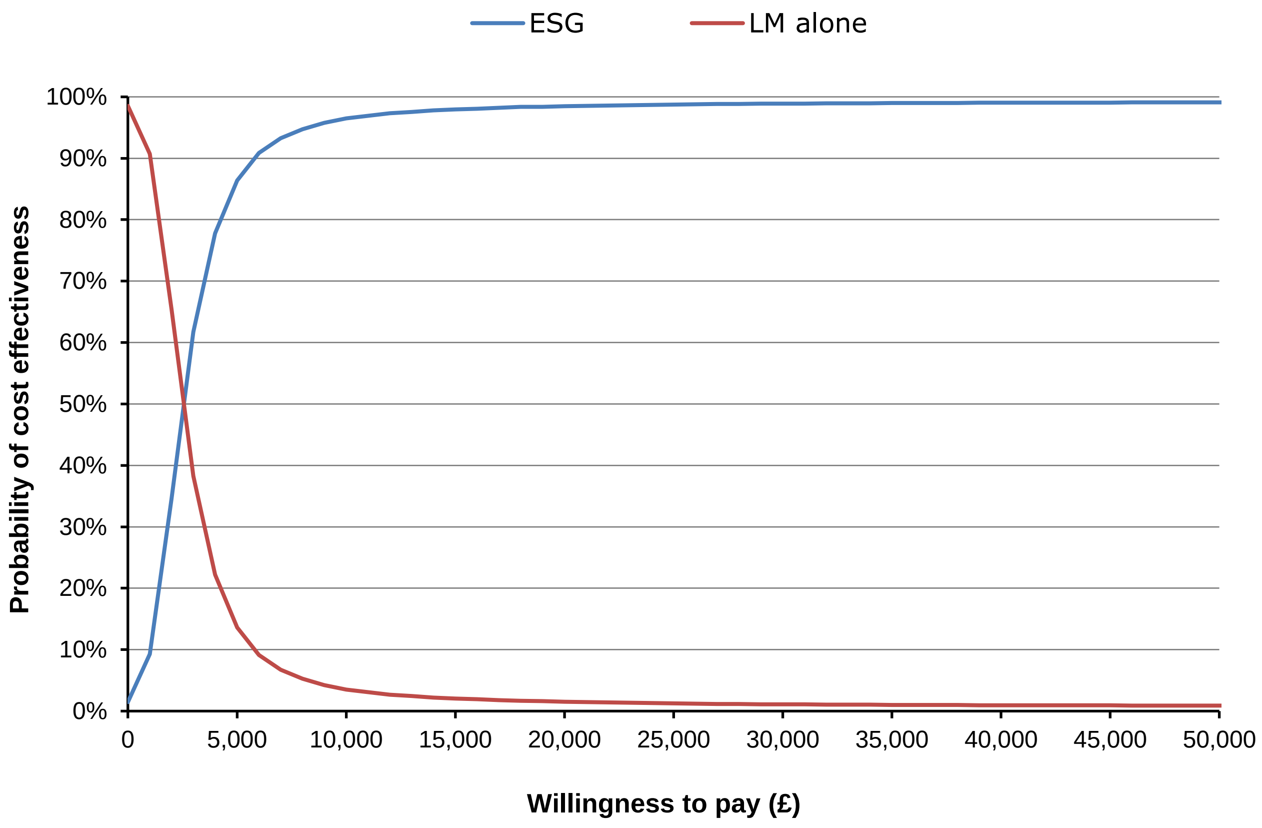

ESG endoscopic sleeve gastroplasty, *LM* lifestyle modification.

References

1. Husereau D, Drummond M, Augustovski F, de Bekker-Grob E, Briggs AH, Carswell C *et al.* Consolidated Health Economic Evaluation Reporting Standards 2022 (CHEERS 2022) statement: updated reporting guidance for health economic evaluations. *BMJ* 2022; **376:** e067975.

2. Apollo Endosurgery Inc. MERIT trial patient-level data. Data on File. 2022.

3. Rowen D, Brazier J, Roberts J. Mapping SF-36 onto the EQ-5D index: how reliable is the relationship? *Health Qual Life Outcomes* 2009; **7:** 27.

4. Ara R, Brazier J. Deriving an algorithm to convert the eight mean SF-36 dimension scores into a mean EQ-5D preference-based score from published studies (where patient level data are not available). *Value Health* 2008; **11**(7)**:** 1131-43.

5. Stephenson J, Smith CM, Kearns B, Haywood A, Bissell P. The association between obesity and quality of life: a retrospective analysis of a large-scale population-based cohort study. *BMC Public Health* 2021; **21**(1)**:** 1990.

6. Wall H, Smith C, Hubbard R. Body mass index and obstructive sleep apnoea in the UK: a cross-sectional study of the over-50s. *Prim Care Respir J* 2012; **21**(4)**:** 371-6.

7. Vusirikala A, Thomas T, Bhala N, Tahrani AA, Thomas GN, Nirantharakumar K. Impact of obesity and metabolic health status in the development of non-alcoholic fatty liver disease (NAFLD): A United Kingdom population-based cohort study using the health improvement network (THIN). *BMC Endocr Disord* 2020; **20**(1)**:** 96.

8. Jacobson BC, Somers SC, Fuchs CS, Kelly CP, Camargo CA, Jr. Body-mass index and symptoms of gastroesophageal reflux in women. *N Engl J Med* 2006; **354**(22)**:** 2340-8.

9. Jones K, Burns A. Unit Costs of Health and Social Care 2021, Personal Social Services Research Unit, University of Kent, Canterbury. In, 2021.

10. NHS England. 2020/21 National Cost Collection Data Publication. 2022. https://www.england.nhs.uk/publication/2020-21-national-cost-collection-data-publication/.

11. National Institute of Health and Care Excellence. Committee Papers. In: Liraglutide for managing overweight and obesity. Technology appraisal guidance [TA664]. 2020. https://www.nice.org.uk/guidance/ta664/evidence. Accessed 18 November 2022.

12. British National Formulary. Enalapril maleate medicinal forms. 2022. https://bnf.nice.org.uk/drugs/enalapril-maleate/medicinal-forms/

13. British National Formulary. Lisinopril medicinal forms. 2022. https://bnf.nice.org.uk/drugs/lisinopril/medicinal-forms/

14. British National Formulary. Perindopril maleate medicinal forms. 2022. https://bnf.nice.org.uk/drugs/perindopril-erbumine/medicinal-forms/.

15. British National Formulary. Ramipril medicinal forms. 2022. https://bnf.nice.org.uk/drugs/ramipril/medicinal-forms/.

16. Xu H, Cupples LA, Stokes A, Liu CT. Association of Obesity With Mortality Over 24 Years of Weight History: Findings From the Framingham Heart Study. *JAMA Netw Open* 2018; **1**(7)**:** e184587.
